# Performance and Reaction Mechanism of a Novel Coking Binder Obtained from Low-Grade Coal

**DOI:** 10.1021/acsomega.4c08311

**Published:** 2024-11-05

**Authors:** Dawei Hu, Zhenzhong Hu, Xian Li, Xianzhe Liu, Zhuoran Chen, Xiaoyong Zhang, Guangqian Luo, Hong Yao

**Affiliations:** † State Key Laboratory of Coal Combustion, School of Energy and Power Engineering, Huazhong University of Science and Technology, Wuhan 430074, China; ‡ Key Laboratory of Coal Clean Conversion and Chemical Process Autonomous Region, School of Chemical Engineering and Technology, Xinjiang University, Urumqi 830000, Xinjiang, China; § School of Chemistry and Chemical Engineering, Anhui University of Technology, Ma’anshan 243002, Anhui, China

## Abstract

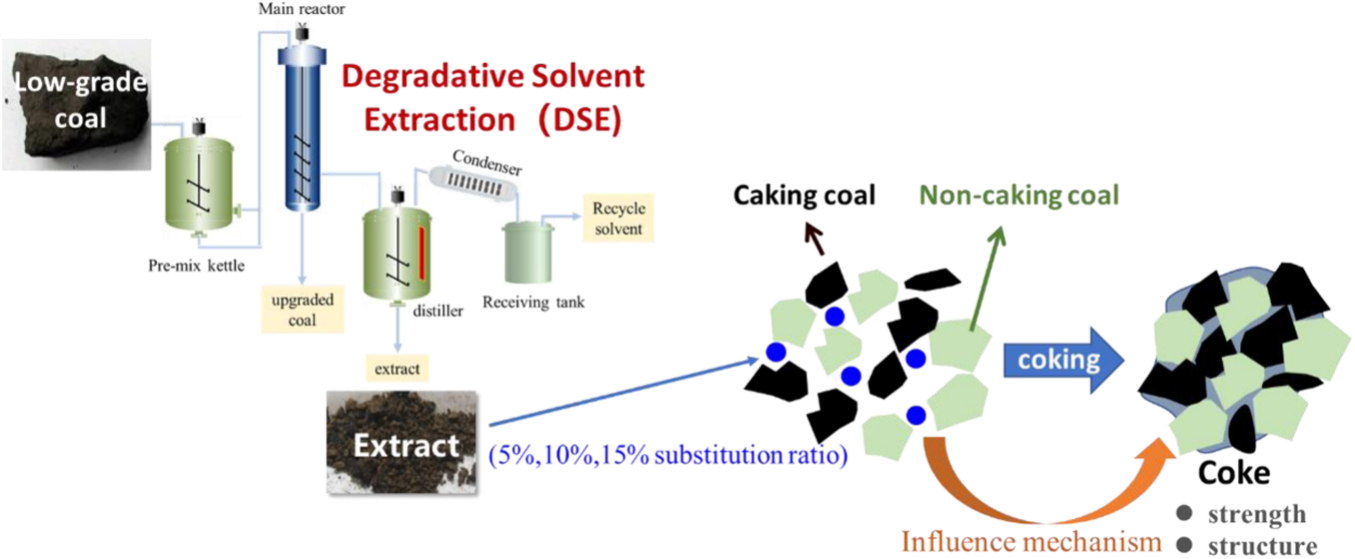

Under the premise of ensuring the quality of coke, reducing coking/fat coal utilization is an urgent problem in the coking industry. This study prepared an extract with softening and bonding characteristics from noncaking low-grade coal by a degradative solvent extraction method. The substitution effect of coking/fat coal by the extract for coking was studied, and the effect mechanism was analyzed in detail. The results show that the caking index of the extract reached 100 and the vitrinite content reached 80%. When the extract substitution ratio is 10%, the coke quality is basically unchanged. Especially when the substitution ratio is 5%, all of the properties of coke are improved, the particle coke reactivity index (PRI) decreases by 4%, the particle coke strength after reaction (PSR) increases by 5%, and the coke microstrength index (MSI) increases by 12%. The influence mechanism model of extract addition on the strength and structure of coke was proposed. The lower softening point of the extract can broaden the plastic interval and improve the caking properties and fluidity of the blended coal, reduce the porosity of coke, and improve the order degree of the carbonaceous structure. Moreover, the highly reactive extract undergoes cross-linking reactions with coal during coking; thus, the cross-link bonds further improve the strength of the coke.

## 1 Introduction

As the world’s largest coke producer, the annual coke production reached 493 million tons in China in 2023, equivalent to caking coal, e.g., coking coal and fat coal, consumption of over 300 million tons.^1^ However, the identified reserves of caking coal in China take up only 13.1% of the total reserves.^2^ Consequently, the massive yearly consumption and relatively scarce reserve of caking coal leads to the vital need for more efficient coke-making technologies.^3,4^ How to reduce the consumption of coking/fat coal while maintaining the coke quality is essential for the coke-making industry.

Based on the coal blending technology, the additive for coal blending, especially the binder, is introduced to improve coke properties by the addition of thermoplastic substances to minimize the need for caking coal.^5−8^ Fernández et al.^5^ proved that the addition of 10% bitumen can reduce 5% of coking coal needed while maintaining similar coke quality. Nomura et al.^9^ found that the addition of 10% coal bitumen to blending coal can significantly increase the DI_15_^150^ of coke by 7%, and Collin et al.^10^ found that the addition of coal bitumen can broaden the plastic temperature range of blending coal. These results clarify the contribution of bitumen to the strength of prepared cokes. However, the high sulfur content adversely affects the quality of coke, and the high cost further diminishes the economic viability of using coal bitumen for coke-making. For economic considerations, some researchers employed other torrefied/upgraded biomass, waste tires, coal gasification slag, and waste plastics as coking additives,^6,11,12^ but their poor thermoplasticity and high ash content resulted in the poor properties of the coke. Zhao et al.^13^ examined the influence of the addition of hypercoal prepared by mixing coal and biomass on the strength of coke, indicating that the optimal blending ratio of hypercoal is 15%, and the compressive strength of coke is 7.76 MPa. However, hypercoal technology has not been industrialized; thus, it cannot guide practical production in the coke industry.

A degradative solvent extraction method (DSE) was proposed to convert low-grade coals into extract with thermoplasticity under mild conditions.^8,12,14^ The method is based on low calorific value, high moisture, high ash, and noncaking coal as raw material through selectively extracting the organic components in the coal to obtain high-carbon, ash-free, and thermoplastic extract by nonpolar organic solvent at about 350 °C.^15,16^ Due to the utilization of low-grade coal as the feedstock and the application of a relatively low processing temperature, this method is a low-cost and simple operational process. At present, the extract has been proven to be a high-quality substitute for coking/fat coal and can reduce the cost of coal blending coking.^17,18^ However, coal blending in coking plants is generally based on experience, with high complexity and diversity. The coal blending scheme with extract addition (such as the type and proportion of the coking coal or fat coal replaced by the extract) has not been systematically studied on the effect of the performance of coke and the reaction mechanism of extract with coal during the coke-making process is also unclear.

Therefore, the extracts were obtained from two kinds of low-grade coal by a pilot system of degradative solvent extraction. The effect of extract substitution of coking/fat coal in blended coal on the coke properties was explored with a substitution ratio from 0 to 15%. The reaction mechanism of the extract with coal during the coke-making process was then studied in detail.

## 2 Materials and Methods

### 2.1 Materials

Two distinct types of low-grade coals, designated as XM and XJ coals, were chosen from the Xiaoma and Xinjiang regions of China for the experiments. XM coal is characterized by elevated levels of ash and sulfur, whereas XJ coal features low ash and sulfur content along with high volatile content and reactivity. Prior to testing, both coals were sieved to a particle size range of 0.15–0.20 mm and thoroughly dried at 105 °C. A cost-effective industrial compounding solvent was used for the extraction, which consisted of 90% two-ring aromatic nonpolar organics and 10% heterocyclic polar matter.

### 2.2 Degradative Solvent Extraction

**Figure 1:**
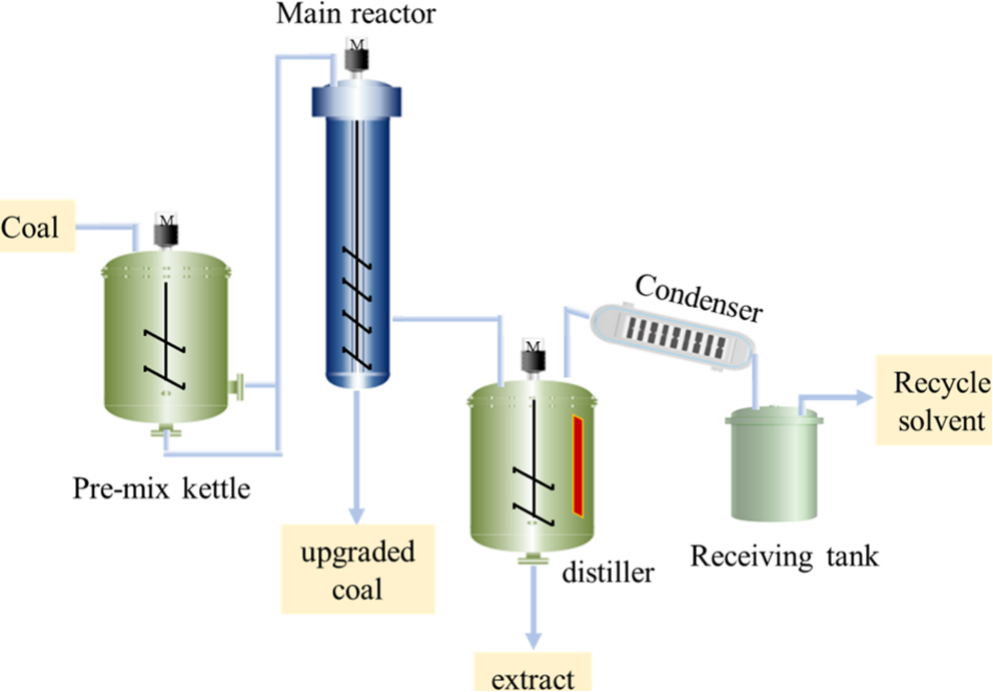
Pilot system of degradative solvent extraction.

The experiment was carried out using a pilot system of degradative solvent extraction, depicted in Figure 1, which mainly included four parts: the premix system, reaction system, separation system, and solvent recovery system. A mixture of 180 kg coal and 900 kg solvent was introduced into the premixing kettle, where it was thoroughly stirred for complete premixing. Subsequently, the mixture was pumped into the main reactor, heated to 360 °C at a consistent rate of 3 °C/min, and held at this temperature for 2 h. The valve under the main reactor was then opened and the liquid went into the distiller through the filter in the main reactor under pressure. The unextracted fraction, which is the upgraded coal, stayed in the main reactor. The solvent was distilled out from the distiller into the receiving tank for recycling, and the remaining solid product in the distiller is denoted as an extract.

### 2.3 Coke Preparation and Evaluation

Five coals were selected and used for the coal blending, including coking coal, fat coal, gas coal, lean coal, and 1/3 coking coal. The proximate analysis and caking index of the coals are shown in Table 1. It is evident that the ash contents in both coking coal and fat coal are more than 10%, and the sulfur contents are also higher than 1%. The higher ash and sulfur contents have an adverse impact on the coke quality. The caking index (*G*) of coking coal and fat coal is more than 80%, which is crucial for the formation of high-quality coke.

**Table 1:** Proximate Analysis and Caking Index of Raw Coals

sample	*A*_d_	*V*_daf_	FC_daf_	*S*_t,d_	*G*
coking coal	10.3	23.2	76.8	1.4	81.7
fat coal	10.8	32.1	67.9	1.1	86.3
gas coal	8.3	38.4	61.6	0.7	55.6
lean coal	11.2	15.8	84.2	0.5	
1/3 coking coal	9.1	32.8	67.2	0.9	72.5

The mixing ratios of the coal in blended coal are shown in Table 2. The proportion of high-caking coals, namely, coking and fat coal, was 50%. Additionally, the substitution ratio of the extracts for coking/fat coals ranged from 5 to 15%. C and F represent coking coal and fat coal, respectively. For instance, XM-C-5% represents replacing 5% of the coking coal with the XM extract. RC acts as the reference blended coal without extract addition.

**Table 2:** Mixing Ratios of the Coal in Blended Coal (*g*)

	sample	coking coal	fat coal	gas coal	lean coal	1/3 coking coal
1	RC	60	40	40	20	40
2	XM-C-5%	57	40	40	20	40
3	XM-C-10%	54	40	40	20	40
4	XM-C-15%	51	40	40	20	40
5	XM-F-5%	60	38	40	20	40
6	XM-F-10%	60	36	40	20	40
7	XM-F-15%	60	34	40	20	40
8	XJ-C-5%	57	40	40	20	40
9	XJ-C-10%	54	40	40	20	40
10	XJ-C-15%	51	40	40	20	40
11	XJ-F-5%	60	38	40	20	40
12	XJ-F-10%	60	36	40	20	40
13	XJ-F-15%	60	34	40	20	40

The crucible coke experiment serves as the coke-making experiment. The blended coal was loaded into a graphite crucible (φ50 mm, L100 mm), followed by the insertion of an iron lump (φ49.5 mm, L70 mm) to apply a pressure of 8 kPa on the coal mixture. Subsequently, the crucible was positioned inside a tube furnace and heated to 1050 °C at a heating rate of 5 °C/min under an Ar gas atmosphere. The temperature was held steady at 1050 °C for 1 h.

### 2.4 Characterization of Raw Coals and the Extracts

The proximate analysis of both raw coals and extracts followed the guidelines outlined in GB/T 212-2008. Ultimate analysis was conducted by using a Vario Micro cube analyzer (Elementar, Germany). The caking index (*G*) was employed to assess the coal’s caking properties, following the guidelines of the National Standard of China (GB5447-85) based on the Roga index.^19^

The FTIR spectral characterization of both raw coals and extracts was conducted using a NICOLET6700 FTIR spectrometer with a resolution of 4 cm^–1^. Samples were prepared by combining 1 mg of the sample with 100 mg of KBr, which was then pressed into pellets.

The maceral components and reflectance of coals were determined by an HD automatic microscope photometer and LEICA lens, according to the determination methods and steps in GB/T 6948-2008 and GB/T 8899-2013.

The softening and melting behavior of the extract was obtained on a hot stage equipped with a microscope. A small piece (about 2 mm × 2 mm) was put into a quartz crucible (diameter 10 mm) on the hot stage (Instec-HCS621G) and protected by high-purity N_2_. The extract was heated from room temperature to 550 °C at a heating rate of 5 °C/min. The softening and melting behavior of the extract with temperature were observed in real-time under a microscope (ZEISS Scope.A1).

### 2.5 Characterization of Cokes

The reactivity toward CO_2_ of the coke was assessed by using a heating furnace, following the coke industry standard (GB/T 4000-2017). The reactivity toward CO_2_ of the coke was measured according to the coke industry standard (GB/T 4000-2017) by a heating furnace. Twenty grams of coke, ranging in size from 3 to 6 mm, underwent a reaction with CO_2_ at 1100 °C for 2 h at a flow rate of 150 mL/min. The weight loss percentage following the reaction defines the particle coke reactivity index (PRI). After the reaction, the drum test was conducted, and the particle coke strength after reaction (PSR) was expressed as the percentage of the coke with a diameter greater than 10 mm after the reaction.^20^ These experiments were conducted in replicates, with errors remaining within a 3% margin.

The microscopic strength characterization of the coke was performed by a self-made microstrength tester. Two grams of coke within a diameter of 0.6–1.25 mm was poured into the steel pipe of the drum device, and 12 steel balls with a diameter of 8 mm were also placed. The test was conducted with a pipe rotation of 800 times at 25 ± 0.5 r/min. After the test, the coke was poured out and the mixture was sieved. The mass ratio of coke samples with particle size greater than 0.2 mm after and before drum rotation is the microscopic strength index of coke (MSI).^21^

The optical textures of coke were determined by the ZEISS Imager M1m optical microscope and the type of optical textures was defined according to YB/T 077-2017 standard.^22^ The optical texture index (OTI) of coke is an index that characterizes the degree of optical anisotropy of coke under a microscope and is calculated by eq 1
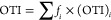
1where *f*_*i*_ is an assignment that represents the degree of anisotropy of the optical microstructure of coke, (OTI)_*i*_ represents the percentage of the optical microstructure of coke.

The Raman spectrum of the samples was analyzed using a high-resolution Raman spectrometer (France).^23^ The measurements were conducted at 25 °C, focusing on the spectral range from 1000 to 1800 cm^–1^. Characterization of the pore structure of coke was performed by using scanning electron microscopy (SEM) images.

## 3 Results and Discussion

### 3.1 Physicochemical Properties of Raw Coals and Extracts

#### 3.1.1 Proximate and Ultimate Composition

Table 3 displays the proximate and ultimate analysis results of the raw coals and their extracts. The ash contents of extracts are significantly reduced to 0.8–1.3% from 7.5 to 40.4% of the raw coals, indicating that more than 95% of total minerals are removed from raw coals. Moreover, the ash contents of the extracts are less than 1/10 of that of coking/fat coals. The ash particles cause a large number of microcracks in the pore walls of coke, thereby reducing its mechanical strength.^24^ Therefore, the extremely low ash content of the extract is an obvious advantage for coking/fat coal replacement in coking. Meanwhile, two types of extracts show high-caking indexes of 100.6 and 98.9, which are higher than those of high-quality coking/fat coal.^7^

**Table 3:** Proximate and Ultimate Analyses of Raw Coals and Extracts

	ultimate analysis (wt %, db)						proximate analysis (wt %, db)			
sample	C	H	N	S	O^a^	H/C	VM	FC^a^	ash	*G*
XM	44.4	3.1	1.0	5.8	5.3	0.84	20.8	38.8	40.4	
XM-Ex	84.7	5.0	2.6	2.1	4.3	0.73	49.5	51.5	1.3	100.6
XJ	74.0	4.7	1.7	0.8	11.3	0.76	38.5	53.0	7.5	
XJ-Ex	86.0	4.9	2.3	0.5	5.5	0.68	58.7	40.5	0.8	98.9

a Calculated by difference.

**Figure 2:**
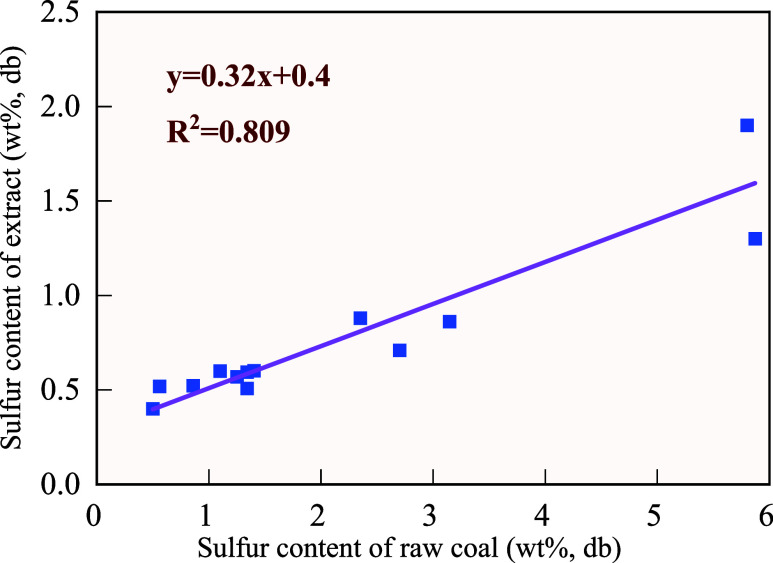
Relationship of the sulfur content between raw coals and extracts.

Table 3 shows that the fuel qualities of extracts were well upgraded by DSE in all indices. The carbon content of both extracts increased, while the oxygen content decreased. Notably, the XM extract exhibited an 86% increase in carbon content, whereas the XJ extract showed a 73% decrease in oxygen content compared to their respective raw coal. The H/C atomic ratio of the extracts is lower than that of the raw coals, indicating that ICS extracts more aromatic components from the coal and causes them to enter the extract. In addition, the sulfur content of the extract is also significantly reduced compared to that of the raw coal. The sulfur content reduction is mainly due to the removal of inorganic sulfur, and only part of organic sulfur migrates to the extract.^25^ It is well-known that the sulfur component in coke is unfavorable to the steel industry. E.g., the sulfur content in coke increases by 0.1%, the coke ratio increases by about 1.5%, the blast furnace production capacity is reduced by 2–2.5%, the amount of limestone is increased by about 2%, and the quality of iron is also reduced.^26^ The sulfur content of the XM extract is higher than that of coking/fat coals, which is caused by the extremely high sulfur content of the raw XM coal, while the sulfur content of XJ extract is about 1/3 of that of coking coal. To reflect the corresponding relationship of sulfur contents between raw coals and extracts, more data were collected from our pilot experiments and relevant ref (27), presented in Figure 2. Overall speaking, the sulfur contents of the extracts are 1/2–1/3 of those of the raw coals. Therefore, the substitution of coking/fat coal by extract for coal blending contributes to the reduction of the sulfur content of the prepared coke.

#### 3.1.2 Softening Properties of the Extract

**Figure 3:**
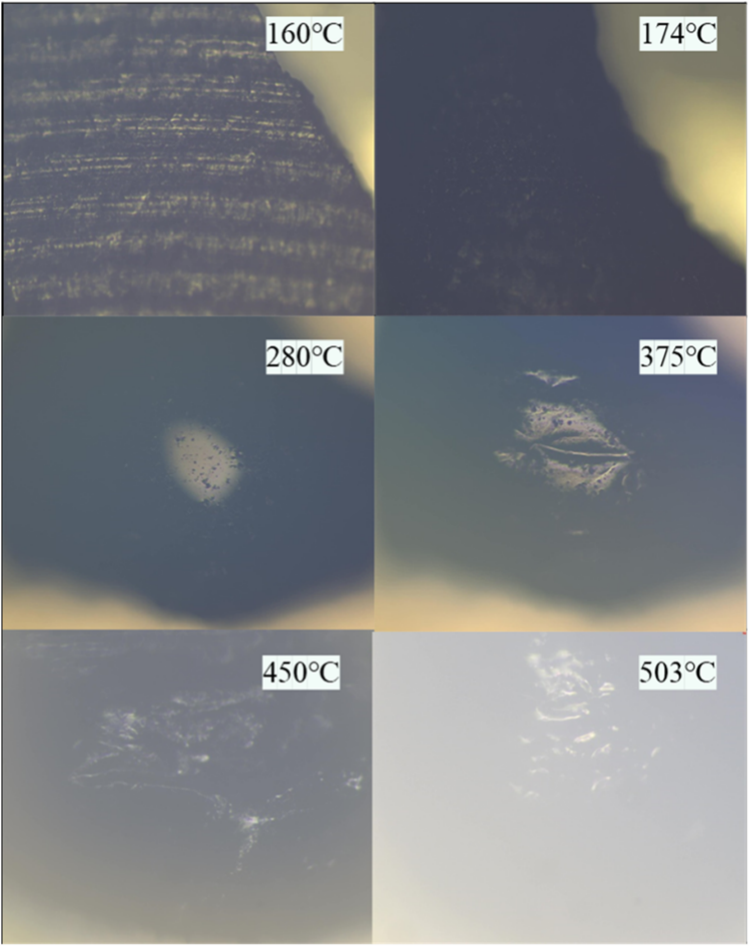
Morphological change of XM extract with temperature under the microscope.

Our previous studies have shown that the extracts derived from various raw coals using DSE technology exhibit highly similar physicochemical properties.^28^ Moreover, the *G* values of the two extracts are very close, indicating that their softening and melting behaviors are similar. Therefore, the XM extract was selected to show its morphological change with temperature, and the results are shown in Figure 3. The extract remained stable before 160 °C, and the bright zone is the reflection of the extract owing to its crystal structure. It started to soften at about 174 °C, and the bright zone disappeared, indicating the phase change. When the temperature increased to around 280 °C, the sample melted mostly. The sample began to solidify at around 375 °C and fully resolidified at around 450 °C. Further increment of temperature exhibited a slight impact on the morphology of the extract. It is well-known that the softening characteristics of coking/fat coal at high temperatures are essential for coking.^29^ The softening temperature of coking/fat coal is generally 350–450 °C.^30^ If the extract was added in blended coal, the temperature range for thermoplasticity in the blended coal can be broadened and the fluidity and caking properties of blended coal can be strengthened.

#### 3.1.3 Chemical Structure of Raw Coal and Extract

**Figure 4:**
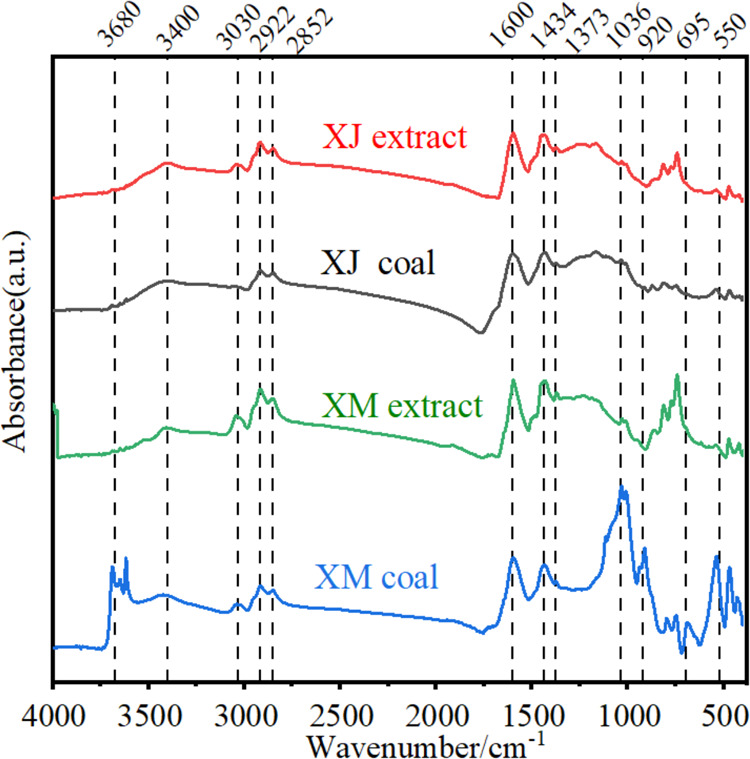
FTIR spectra of both raw coals and their extracts.

Figure 4 displays the FTIR spectra of both raw coals and their respective extracts. The spectra reveal significant differences in the five main types of functional groups between the coals and extracts. These include mineral matter, observed around 3600–3700 and 500–600 cm^–1^, aromatic C–H groups, around 3030 and 920–695 cm^–1^, aliphatic −CH_2_ and −CH_3_ groups, around 2852 and 2922 cm^–1^, aromatic C=C bonds, noticeable at 1600 cm^–1^, and C–O around 1036 cm^–1^.^[Bibr ref]^

First of all, the chemical structures of the extracts obtained from the two kinds of raw coals with very different chemical structures are basically similar, which indicates the homogenizing effect of DSE for the coals. Compared to the raw coals, the peaks of extracts belonging to minerals almost disappeared, mostly indicating their removal. The relative intensity ratio in the FTIR spectrum can indicate the structural change of coal. The absorption peak representing aromatic C–H in the extract obviously increases, and the aliphatic CH_2_ and CH_3_ peaks were also enhanced slightly, indicating the thermal dissolution of aromatic compounds and aliphatic compounds into the extract from coal. Compared with raw coal, there is no obvious C–O stretching vibration characteristic peak in the extract, indicating their effective breakage during DSE. Thus, it can be inferred that the small and medium molecular aromatic components and aliphatic components were extracted by destroying the cross-linking bonds of chemical structure in raw coal, such as the C–O–C bond, during the DSE process.

#### 3.1.4 Maceral Composition of the Extract

The maceral components of the XM and XJ extracts, shown in Table 4, are mainly vitrinite, which reached 85.4 and 78.5%, respectively. Their active components are 85.7 and 79.9%, respectively, and the inertinite concentrations are as low as 12.7 and 19.4%. The vitrinite content of the extract is significantly higher than that of coking/fat coal, which is generally between 60 and 75%. During the coking process, vitrinite evolves into a plastic mass that either adheres to itself or combines with inert substances, playing a gelatinizing role, which is essential for coking.^32^

**Table 4:** Maceral Composition of Extracts (wt %)

	active component			inert component		
sample	vitrinite	exinite	total	inertinite	mineral	total
XM-Ex	85.4	0.3	85.7	12.7	1.6	14.3
XJ-Ex	78.5	1.4	79.9	19.4	0.7	20.1

**Figure 5:**
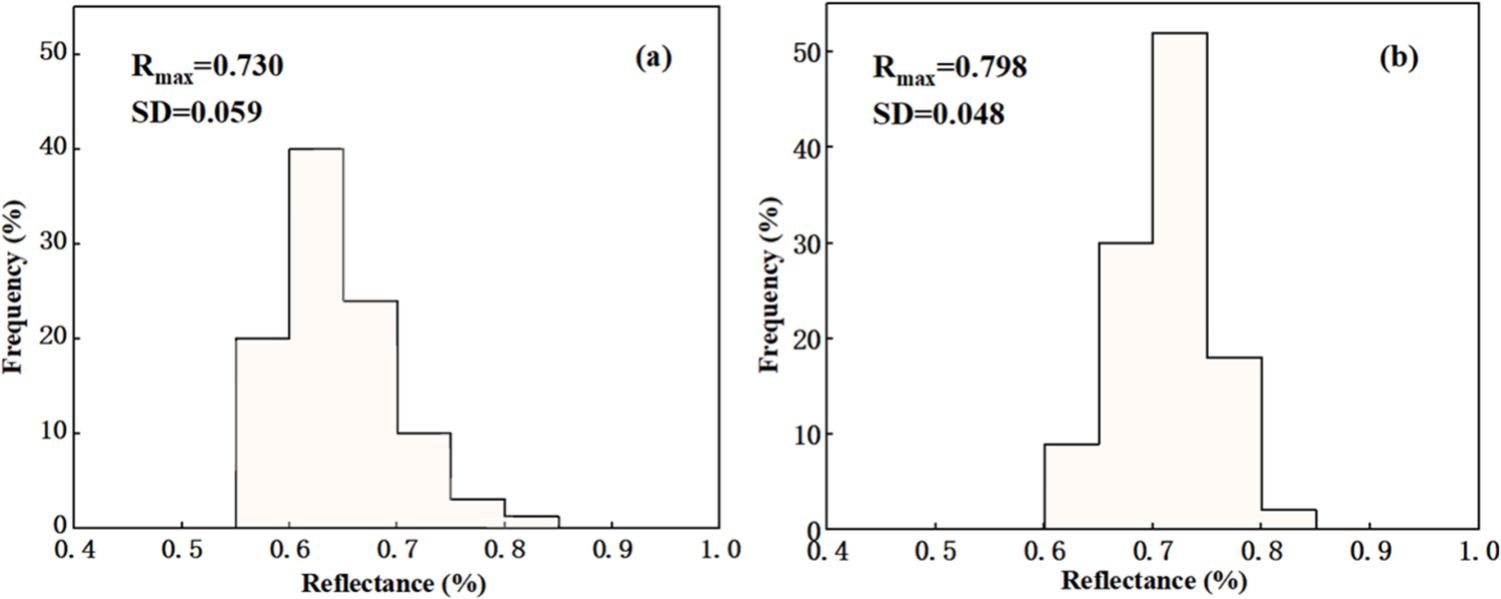
Vitrinite reflectance distribution of extracts: (a) XM and (b) XJ.

The distributions of vitrinite reflectance of the two extracts, as shown in Figure 5, are concentrated within 0.55–0.85, but their specific distribution intervals are different. The main distribution interval of XM extract vitrinite ranges from 0.6 to 0.75, while that of XJ extract ranges from 0.65 to 0.8. Figure 5 also shows the mean maximum reflectance of vitrinite (*R*_max_) and the standard variance of the reflectance distribution (SD) of the two extracts. The *R*_max_ values of the two extracts are 0.730 and 0.798, respectively, indicating that they belong to coal with a low degree of metamorphism. However, the *R*_max_ of lignite is generally less than 0.5, indicating that the metamorphism degrees of extracts are higher than those of the raw coals. The standard variances of the reflectance distribution of extracts were less than 0.1, and the reflectance distribution map is a standard normal distribution with notch. According to the determination method of the reflectance distribution map of commercial coal in GB/T15591-1995, both extracts resemble the single-seam coal. In addition, some scholars^33,34^ found that when the *R*_max_ of coal is between 0.70 and 0.95, the optical structure of the formed coke is dominated by the mosaic structure, which is favorable to the strength of coke. The *R*_max_ values of the two extracts are in this range.

### 3.2 Crucible Coking Quality Evaluation

#### 3.2.1 Coke Yield

**Figure 6:**
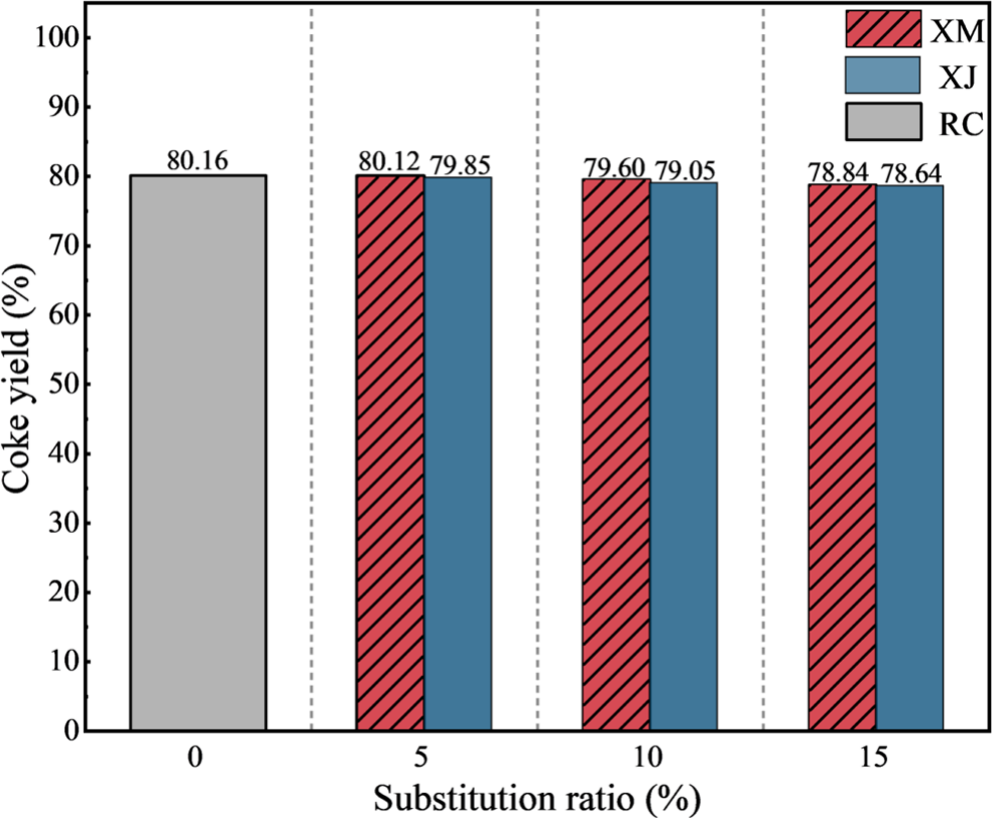
Yield of cokes under different extract substitution ratios.

The yields of cokes under different extract substitution ratios are shown in Figure 6. It is evident that it has no obvious impact on the coke yield, although the volatile content of the extract is significantly higher than that of coking/fat coal. When the substitution ratio reaches 15%, the coke yield only decreases by 1.8%, indicating that the volatiles of the extract do not escape during the coking process but react with blended coal, thus affecting the quality of coke. For the slight yield decrease for the substitution ratio of 15%, this part of the extract was converted to high-temperature coke-oven coal tar and gas, which are also the main products for the current coke-making industry.

#### 3.2.2 Coke Properties and Strength

**Figure 7:**
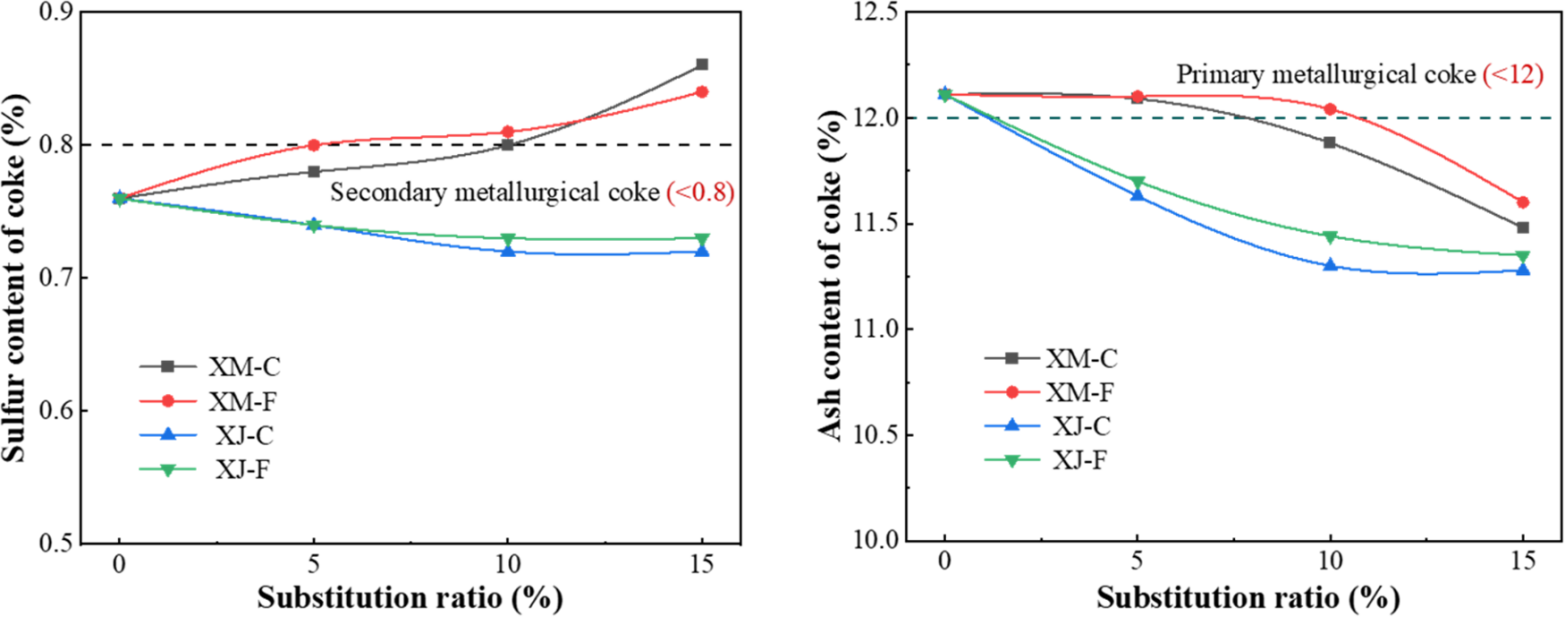
Changes of the sulfur and ash contents in crucible coke.

The variation of sulfur contents and ash contents in crucible coke to the extract substitution ratio is shown in Figure 7. The sulfur content of coke decreases with the addition of the XJ extract. When the substitution ratio for coking coal is 10%, which corresponds to a 3% content in the overall blended coal, the sulfur content of the resulting coke is reduced by 5%. The sulfur content of the coke increases with the level of XM extract substitution, which is due to the high sulfur content of the XM extract. The ash content of coke decreases with the increase of the substitution ratio of the extract. According to the metallurgical coke quality standards (GB/T1996-2003), when the extract replaces coking coal by 10%, the ash content of the coke is reduced by 7% and is raised to the level of the primary metallurgical coke (ash <12%).

**Figure 8:**
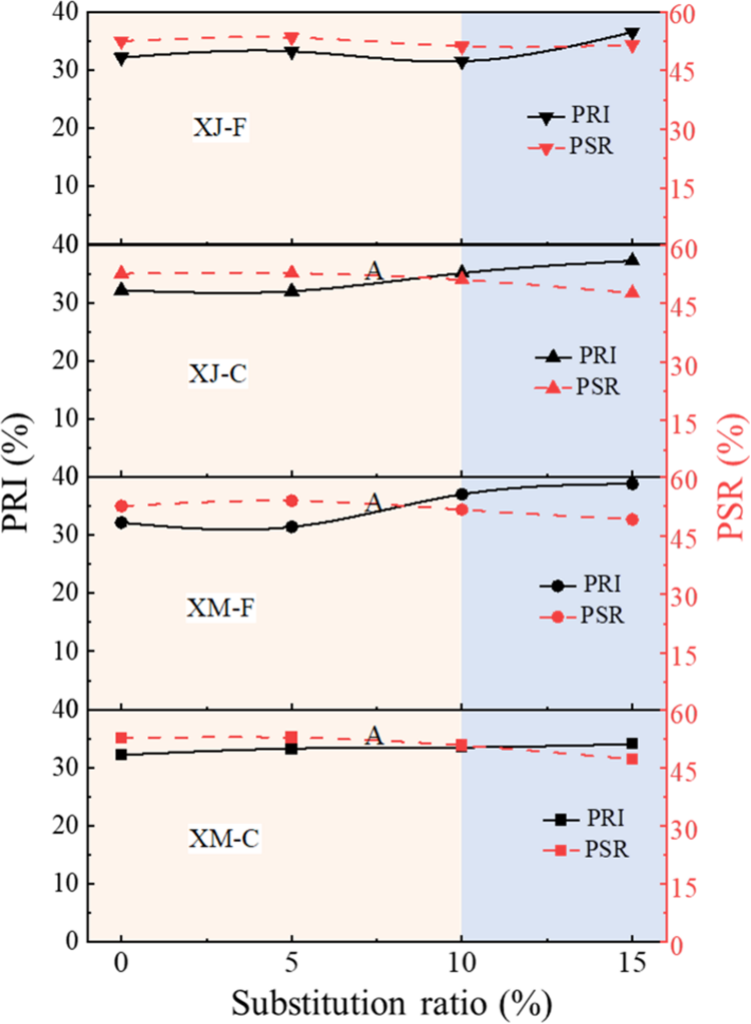
PRI and PSR of cokes under different extract substitution ratios.

The PRI and PSR are the most important parameters for the coke quality evaluation. A smaller PRI implies that coke is less easily consumed and can maintain strength at high temperatures during the steelmaking process.^11^ PSR is the mechanical strength of coke at high temperatures.^29^Figure 8 shows the PRI and PSR values of crucible cokes. When the substitution ratio of the two extracts is 0–10%, the PRI and PSR of the cokes remain stable, with a variation of less than 3%. It is worth mentioning that when the substitution ratio is 5%, the PSR of cokes increases for all samples. When the substitution ratio is 15%, the PSR of cokes decreases slightly, remaining within 5%, while the PRI increases by no more than 8%. Therefore, the substitution ratio of the extract is recommended to be 10%, where the PRI and PSR of cokes still meet the standard of secondary metallurgical coke (PRI < 35%, PSR > 50%).

**Figure 9:**
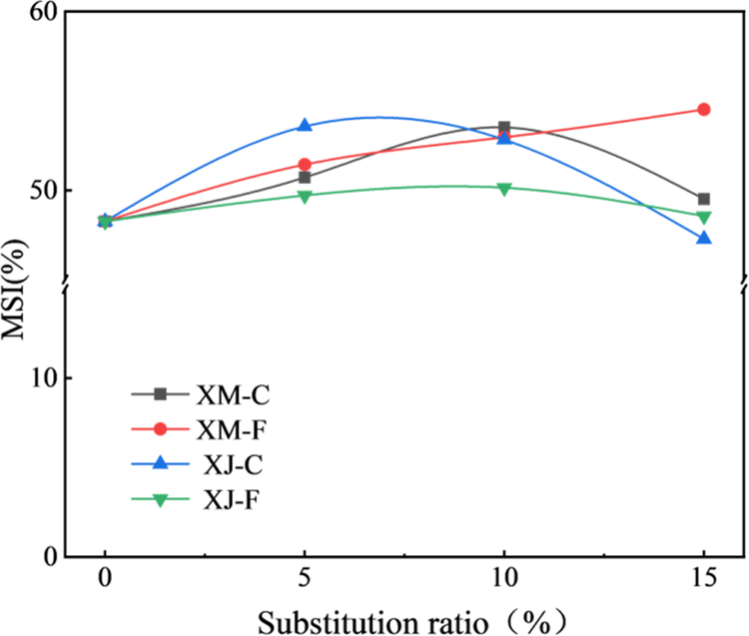
MSI of cokes under different extract substitution ratios.

The MSI reflects the abrasion resistance of the pore wall of coke, which is considered to be an ideal method for the estimation of the pore wall strength of coke.^21^ The particle size of the test sample is relatively small (0.6–1.25 mm); therefore, the influence of porosity is basically excluded. MSI is mainly associated with the pore wall material and the thickness of the pore walls in coke. Some scholars found that it is related to the volatile content in the blended coal.^35^Figure 9 shows the MSI of the crucible cokes. When the substitution ratio is 5 and 10%, the MSI of all cokes increases significantly. The MSI of XJ-C coke with a substitution ratio of 5% is as high as 53.6%, which is 11% higher than that of the reference coke. XJ-C blended coal has the highest volatile content because of the high volatile content of the XJ extract and the low volatile content of coking coal. When the substitution ratio is 15%, the MSI of the cokes starts to decrease, except for XM-F coke, which increases to 54.5%. The volatile content of the blended coal increases with the increase in the extract substitution ratio because of the high volatile content of the extract. However, the XM-F blended coal should have the lowest volatile content compared to the other three blended coals with the same extract substitution ratio of 15%. These imply that the excessively high volatile content of the blended coal is unfavorable to the MSI of the coke, and there is an upper limit of the extract substitution ratio for caking coal in the blended coal. 10% should be the appropriate substitution ratio, as illustrated in Figure 9.

#### 3.2.3 Coke Optical Structure

Table 5 provides the quantitative evaluation results for the optical texture of the cokes. The optical texture of metallurgic coke is categorized into an anisotropic group and an isotropic group. The anisotropic group encompasses fine mosaic, medium mosaic, coarse mosaic, fibrous, and lamellar structures. The isotropic group encompasses basic isotropy and fusinite block. Compared with the reference coke, the total isotropic group of cokes with extract addition slightly increases, but the basic isotropy decreases. The increase of the total isotropic group is mainly due to the increase of the fusinite block. The total anisotropy of coke decreases slightly with the extract addition, but the mosaic structure increases. For example, the medium mosaic structure of XJ-F-10% coke measures 40.0, which is higher than that of the reference coke of 29.3. These indicate that more medium mosaic structures and fusinite-block structures were formed during the coking process with the extract. Due to the chemical bonds within the mosaic structure of the coke, a strong caking force exists, rendering coke with a higher proportion of mosaic structure less prone to fracturing and thereby exhibiting superior cold strength. Additionally, the mosaic structure of coke played an important role in restraining crack propagation and mitigating thermal expansion.^36^ Finally, although the addition of extracts slightly reduces the OTI of coke compared to the reference coke, the reduction remains within 5%. Thus, the replacement of a portion of coking/fat coal by the extract has no significant effect on the optical structure of the coke.

**Table 5:** Optical Structure Content of Cokes (wt %)

	anisotropic group						isotropic group			
sample	fine mosaic	medium mosaic	coarse mosaic	fibrous	lamellar	total	basic isotropy	fusinite-block	total	OTI
RC	16.3	29.3	20.7	5.6	2.1	74.1	16.8	9.1	25.9	141.5
XM-C-5%	11.2	31.6	20.5	5.1	1.7	70.1	14.2	15.6	29.8	136.5
XM-C-10%	13.1	33.1	17.5	7.3	1.3	72.3	14.6	13.1	27.7	141.4
XM-C-15%	14.0	37.1	14.5	4.5	1.0	71.2	15.5	13.3	28.8	134.7
XM-F-5%	12.9	32.1	17.0	5.8	2.4	70.2	17.4	12.4	29.8	134.1
XM-F-10%	15.5	37.4	11.1	6.4	0.7	71.1	13.1	14.8	28.9	132.5
XM-F-15%	19.5	38.6	10.1	4.6	0.5	73.4	11.2	15.4	26.6	140.7
XJ-C-5%	20.6	31.0	13.5	4.5	1.3	74.5	9.2	15.8	25.5	136.5
XJ-C-10%	19.1	34.0	14.6	4.3	1.3	73.2	14.3	12.5	26.8	134.4
XJ-C-15%	16.7	35.1	16.3	7.3	1.3	72.7	15.3	12.0	27.3	138.4
XJ-F-5%	11.1	38.9	15.0	4.8	1.6	71.3	13.4	15.3	28.7	139.7
XJ-F-10%	14.9	40.0	15.5	3.6	0.6	74.7	11.6	13.7	25.3	139.1
XJ-F-15%	14.1	36.7	16.2	4.8	1.2	73.0	11.1	15.9	27.0	136.2

#### 3.2.4 Coke Carbon Structure

**Figure 10:**
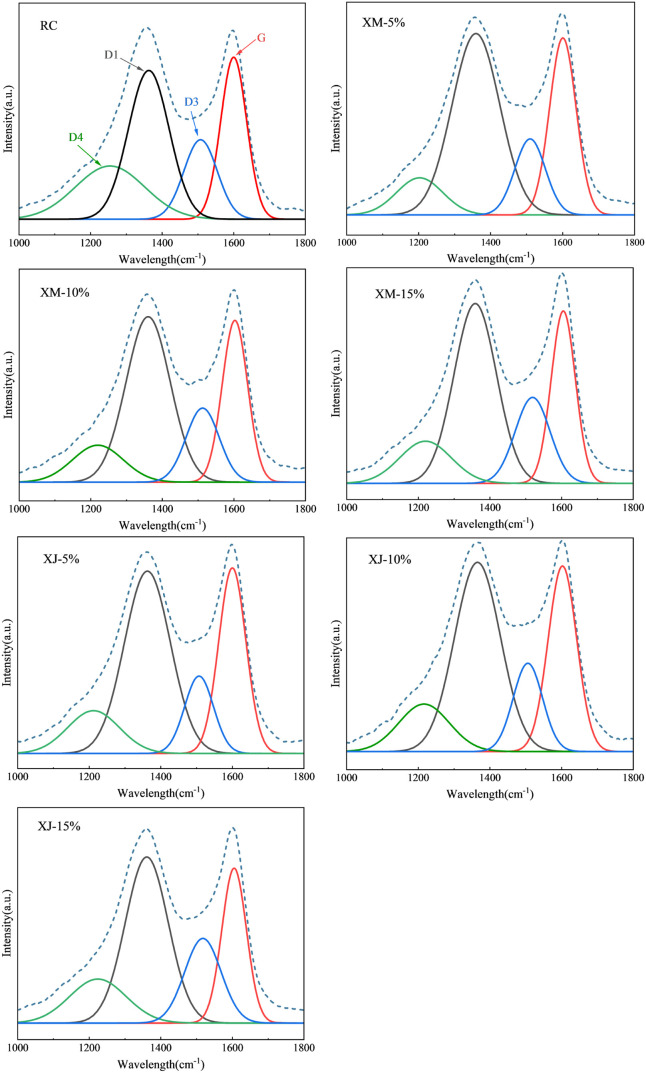
Raman spectrogram of cokes under different extract substitution ratios.

Raman analysis technology was employed to explore the effect of the extract addition on the carbon order degree of the coke. Raman spectra of carbon materials were typically fitted using five peaks with wave numbers between 1000 and 1800 cm^–1^, defined as D4, D1, D3, G, and D2 peaks with wave numbers of 1200, 1360, 1500, 1580, and 1620 cm^–1^, respectively.^37^ However, the D2 peak was not prominently detected in the cokes; therefore, the effect of the D2 peak is not considered, and the remaining peaks were fitted using the Gauss method. The G peak is commonly employed to describe the in-plane stretching vibration of the planar triangular structure resulting from the sp^2^ hybridization of atomic orbitals within graphite frameworks. In the case of carbon-containing materials with an uncertain nature, the D1 peak at 1360 cm^–1^ is used to identify lattice defects within the graphite structure. The Raman spectrum fitting results of cokes are shown in Figure 10. The experimental results of the Raman spectra closely align with the fitting results. However, accurately and quantitatively characterizing the change patterns in the carbonaceous structure among different samples proved challenging solely through the depicted results in the figure. The *A*_G_/*A*_all_ ratio serves as a quantitative measure to characterize the degree of order of the carbon structure. A higher *A*_G_/*A*_all_ value indicates a greater order degree of the carbonaceous structure.^38,39^ The values of *A*_G_/*A*_all_ under different substitution ratios of extracts are presented in Table 6. The *A*_G_/*A*_all_ of the cokes with the substitution ratio was 0–10%, which is higher than that of the reference cokes; especially when the substitution ratio is 5%, the *A*_G_/*A*_all_ of the two cokes reached 0.2699 and 0.2822, respectively. With the further increase of the substitution ratio, the *A*_G_/*A*_all_ of coke is smaller than that of the reference coke. This shows that the carbonaceous order degree of coke first increases and then decreases with the increase of the extract substitution ratio, which is consistent with the changes in the cold and hot strength of the cokes mentioned above.

**Table 6:** Raman Characteristic Parameters of Cokes under Different Extract Substitution Ratios

sample	*A*_G_/*A*_all_
RC	0.2536
XM-5%	0.2699
XM-10%	0.2606
XM-15%	0.2456
XJ-5%	0.2822
XJ-10%	0.2795
XJ-15%	0.2305

#### 3.2.5 Coke Surface Morphology

**Figure 11:**
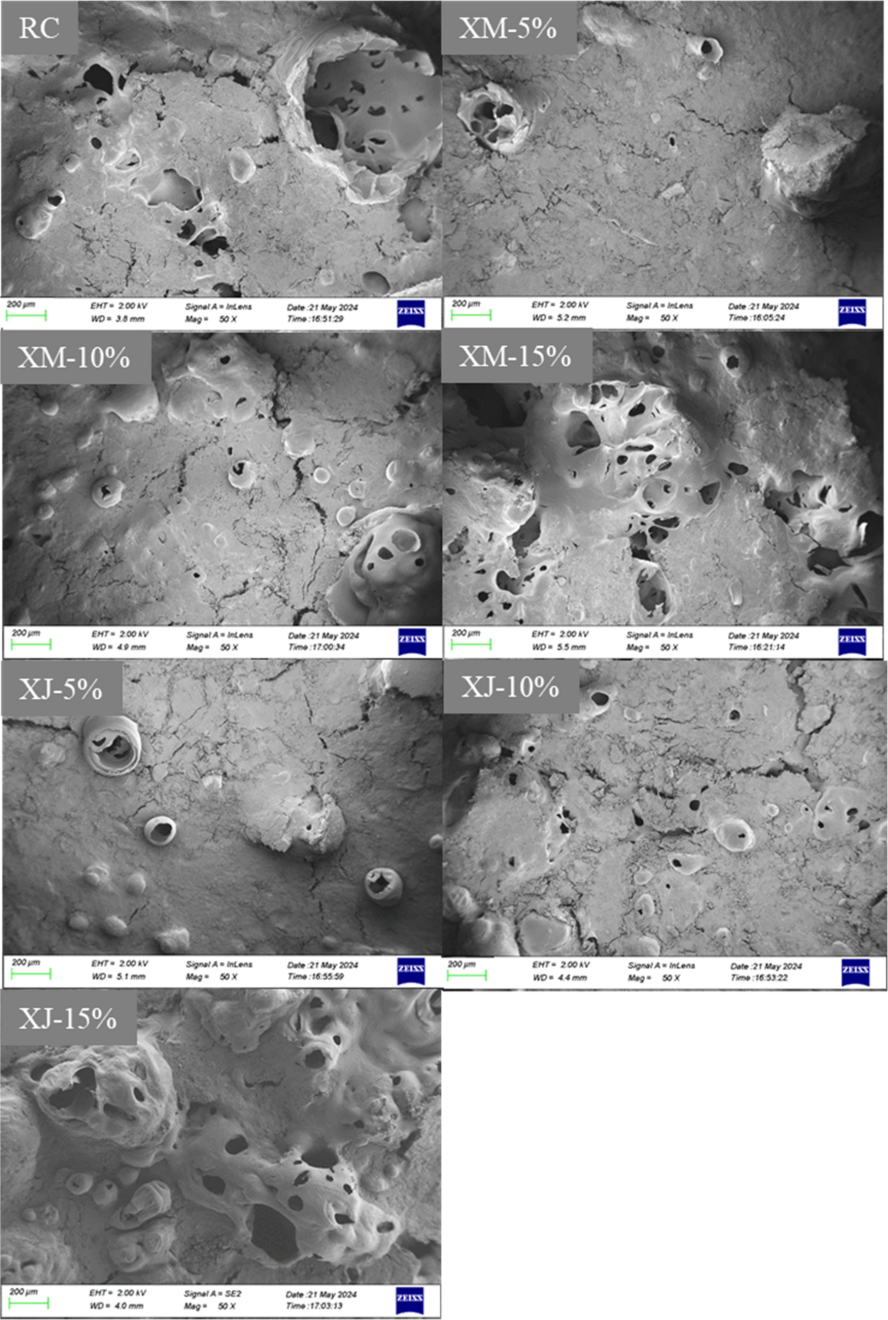
Scanning electron microscopy (SEM) images of the cokes.

The SEM images of the cokes are shown in Figure 11. The number and diameter of pores in the coke with the extract substitution ratio of 5% are obviously smaller than those of the reference coke. These are still similar to those of the reference coke when the substitution ratio is 10%. When the extract substitution ratio reaches 15%, the diameter of the coke pores increases significantly, which is because the volatile content in the blended coal is too high, leading to excessive volatilization during the coking process and resulting in an overly porous coke structure. This is consistent with the previously mentioned variations in coke strength. When the extract substitution ratio is 10%, the PRI, PSR, and MSI of cokes remain largely unchanged. At a 5% substitution ratio, strength increases, whereas at a 15% ratio, strength decreases.

### 3.3 Influence Mechanism of Extract Addition on the Strength and Structure of Coke

**Figure 12:**
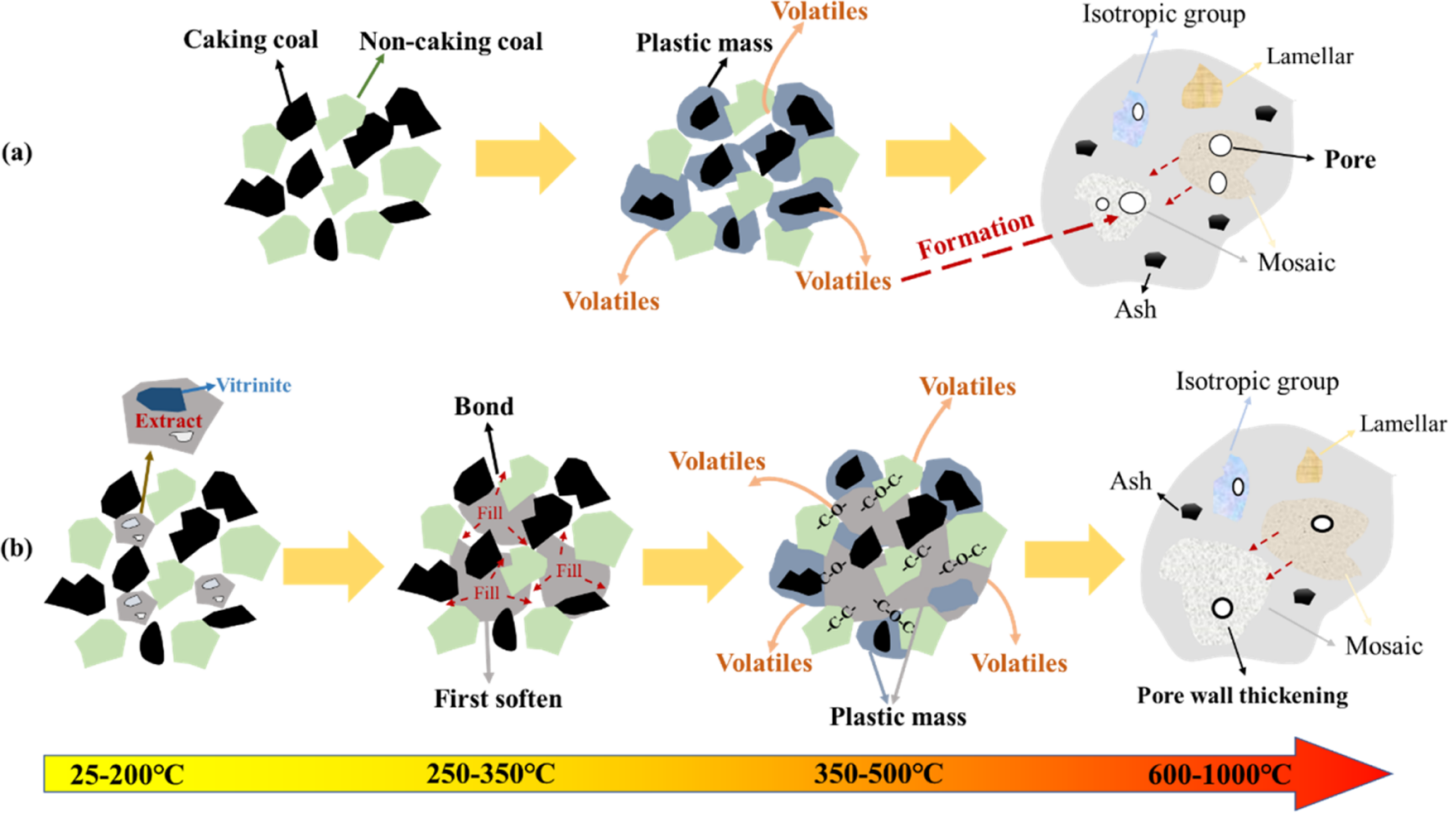
Coking process of blended coal (a) and influence mechanism of extracts (b).

The coking process is categorized into three stages, as shown in Figure 12a, partial vaporization and gas release, softening and resolidification of coal, and shrinkage and solidification of semicoke. The second stage, occurring within the temperature range of 350–550 °C, is the most crucial phase in the coal coking process. During this stage, active components, such as vitrinite, undergo softening and melting, forming a plastic mass that binds other substances upon reaching a certain temperature. This plastic mass flows, expands, and eventually solidifies. The properties and quantity of this plastic mass are crucial factors in determining the quality of coke.

The extract is a product extracted from coal between 350 and 400 °C, a temperature range that closely matches the precipitation temperature range of the plastic mass in caking coal during coking. Moreover, the extract contains more than 80% vitrinite. So, it can be considered that the extract is rather similar to the plastic mass in coal. Additionally, the softening point of the extract is lower than that of coking/fat coal, which can broaden the thermoplastic temperature range of the blended coal. Furthermore, the extract exhibits high activity and readily undergoes cross-linking reactions with coal, achieving a caking index of up to 100, which can improve the chemical connection between coal particles. Besides, its low ash and sulfur contents further enhance the coke quality.

Drawing from the aforementioned theoretical studies, the influence mechanism of extract addition on the structure and strength of coke is proposed and illustrated in Figure 12b. Compared with the caking coal, the extract softens and bonds in advance during the coking process at 250–350 °C, which improves the caking properties and fluidity of the blended coal. This enhances the contact between particles, ensuring the stability of the carbon structure of coke and reducing the number of surface pores. Furthermore, when the temperature rises above 350 °C, the extract undergoes cross-linking reactions with coal to form cross-link bonds, thereby enhancing coke strength after resolidification. From the perspective of the microstructure of the extract, a large number of vitrinite components in the extract plays a softening and bonding role in the coking process and can be attached to the coal. The average maximum reflectance of vitrinite is about 0.75, and more mosaics are formed in the coking process. It can ensure the strength of coke.^6^

## 4 Conclusions

The coke properties remain unchanged when the extract substitution ratio is 10%. Especially at a 5% substitution ratio, coke quality improves with a reduction of PRI by 4%, an increase of PSR by 5%, and MSI by 12%, along with a significant decrease in both the number and diameter of pores. Furthermore, the reaction mechanism model of the extract with coal during coking was established. At 250–350 °C, the extract softens and bonds in advance, thus broadening the plastic interval of the blended coal. When the temperature rises above 350 °C, the highly reactive extract undergoes cross-linking reactions with blended coal, thereby forming cross-linking bonds that increase the coke strength.
